# Supplementary material for: Cell-type-specific control of basolateral amygdala neuronal circuits via entorhinal cortex-driven feedforward inhibition
Source: eLife. 2020 Jan 9;9:e50601. doi: 10.7554/eLife.50601 (PMC6984813; doi:10.7554/eLife.50601)
Supplement: Transparent reporting form [file elife-50601-transrepform.docx]

***eLife’s* transparent reporting form**

We encourage authors to provide detailed information *within their submission* to facilitate the interpretation and replication of experiments. Authors can upload supporting documentation to indicate the use of appropriate reporting guidelines for health-related research (see [EQUATOR Network](http://www.equator-network.org/%20)), life science research (see the [BioSharing Information Resource](https://biosharing.org/)), or the [ARRIVE guidelines](http://www.plosbiology.org/article/info:doi/10.1371/journal.pbio.1000412) for reporting work involving animal research. Where applicable, authors should refer to any relevant reporting standards documents in this form.

If you have any questions, please consult our Journal Policies and/or contact us: [editorial@elifesciences.org](mailto:editorial@elifesciences.org).

**Sample-size estimation**

- You should state whether an appropriate sample size was computed when the study was being designed
- You should state the statistical method of sample size computation and any required assumptions
- If no explicit power analysis was used, you should describe how you decided what sample (replicate) size (number) to use

Please outline where this information can be found within the submission (e.g., sections or figure legends), or explain why this information doesn’t apply to your submission:

Details on we chose sample size were not included in the submission, but will be included in the “Quantification and Statisical Analysis” subsection of the “Materials and Methods” section of the manuscript when revisions are made following reviewer comments.

No formal power analysis was made as we had no *a priori* expectation for effect sizes.

Replicate size was a minimum of three separate mice for each experimental condition. When fewer replicates occurred due to experimental constraints (see lines 241, 242, page 11), these data were excluded from formal analyses and are presented as Figure S4 so the reader has access to them.

For all experiments, we strived to maintain equal numbers across conditions.

In experiments comparing PV, FS Sst, and nFS Sst neurons, this was not always possible as we found more FS Sst compared to nFS Sst neurons in our recordings. However, Sst neuron identity was not determined until cluster analyses were run after all data had been collected. As a result for these experiments, we strove to include twice as many Sst neurons as PV neurons to best equalize the number of neurons in each group. For example, Table S1 shows the physiological properties of these three neuronal subtypes. We recorded 52 PV neurons and 105 total Sst neurons (60 were classified as FS and 45 as nFS).

**Replicates**

- You should report how often each experiment was performed
- You should include a definition of biological versus technical replication
- The data obtained should be provided and sufficient information should be provided to indicate the number of independent biological and/or technical replicates
- If you encountered any outliers, you should describe how these were handled
- Criteria for exclusion/inclusion of data should be clearly stated
- High-throughput sequence data should be uploaded before submission, with a private link for reviewers provided (these are available from both GEO and ArrayExpress)

Please outline where this information can be found within the submission (e.g., sections or figure legends), or explain why this information doesn’t apply to your submission:

We did not include a formal definition of biological vs technical replication. However, we will include a formal definition in the “Quantification and Statisical Analysis” subsection of the “Materials and Methods” section of the manuscript when revisions are made following reviewer comments.

For our experiments, our biological replicates were the mice from which we recorded neurons for each experimental condition. Our technical replicates were the number of neurons recorded within each experimental condition.

The number of technical and biological replicates are reported for each experimental condition in both the text of the “Results” section as well as in the figure legends. The replicates are reported as n = x, y where x is the number of technical replicates (neurons) and y is the number of biological replicates (mice). The details of this reporting is already included in the “Methods and Materials” section of the manuscript on lines 1099, 1100 on page 56.

Data were only discarded if (1) access resistance exceeded 25 MΩ or varied by more than $\boldsymbol{\pm}$ 20% (“Materials and Methods” section, lines 812-814, page 44) or (2) if the eEPSC amplitude did not achieve a stable level following plasticity induction during plasticity experiments – this was done to ensure that changes in eEPSC amplitude were related to plasticity induction and were not a confound of other phenomena (details on how stability was determined are included in the “Materials and Methods” section, lines 898-902, page 47).

All other data were included, including potential outliers, as to best capture the variability that occurred within the biological system we studied.

**Statistical reporting**

- Statistical analysis methods should be described and justified
- Raw data should be presented in figures whenever informative to do so (typically when N per group is less than 10)
- For each experiment, you should identify the statistical tests used, exact values of N, definitions of center, methods of multiple test correction, and dispersion and precision measures (e.g., mean, median, SD, SEM, confidence intervals; and, for the major substantive results, a measure of effect size (e.g., Pearson's r, Cohen's d)
- Report exact p-values wherever possible alongside the summary statistics and 95% confidence intervals. These should be reported for all key questions and not only when the p-value is less than 0.05.

Please outline where this information can be found within the submission (e.g., sections or figure legends), or explain why this information doesn’t apply to your submission:

All statistical analyses and justifications for them are described in the “Quantification and Statistical Analysis” subsection of the “Materials and Methods” section of the manuscript on pages 56-59.

Figure legends report the statistical test used, *p* values, and n, as well as describing how summary statistics and measures of dispersion are displayed within the figure. The above and additional details on statistical parameters and tests used are reported in the “Results” section for each experiment we conducted.

(For large datasets, or papers with a very large number of statistical tests, you may upload a single table file with tests, Ns, etc., with reference to sections in the manuscript.)

**Group allocation**

- Indicate how samples were allocated into experimental groups (in the case of clinical studies, please specify allocation to treatment method); if randomization was used, please also state if restricted randomization was applied
- Indicate if masking was used during group allocation, data collection and/or data analysis

Please outline where this information can be found within the submission (e.g., sections or figure legends), or explain why this information doesn’t apply to your submission:

Biological replicates (mice) were randomly selected on the day of experiment and technical replicates (neurons) were randomly selected during experiments.

This was not specifically stated in the original submission of the manuscript but will be included in the revised manuscript in a new “Group Allocation” subsection of the “Materials and Methods” section following reviewer feedback.

**Additional data files (“source data”)**

- We encourage you to upload relevant additional data files, such as numerical data that are represented as a graph in a figure, or as a summary table
- Where provided, these should be in the most useful format, and they can be uploaded as “Source data” files linked to a main figure or table
- Include model definition files including the full list of parameters used
- Include code used for data analysis (e.g., R, MatLab)
- Avoid stating that data files are “available upon request”

Please indicate the figures or tables for which source data files have been provided:

Initial submission of the manuscript states that “Data are available on request”; this statement will be removed and data files will be included with the revised manuscript following reviewer feedback.

Code can be found at <https://github.com/emguthman/Manuscript-Codes> and this is stated on lines 1149, 1150, page 59.
